# A new disjunct *Dendrothrix* (Euphorbiaceae, tribe Hippomaneae): a Guiana Shield element in sub-Andean cordilleras of Ecuador and Peru

**DOI:** 10.3897/phytokeys.86.14761

**Published:** 2017-10-03

**Authors:** Kenneth J. Wurdack

**Affiliations:** 1 Department of Botany, MRC-166, National Museum of Natural History, Smithsonian Institution, P.O. Box 37012, Washington DC 20013-7012, USA

**Keywords:** Cordillera del Cóndor, *Dendrothrix*, Hippomaneae, leaf morphology, seeds

## Abstract

*Dendrothrix condorensis* K.Wurdack, **sp. nov.** from the sub-Andean cordilleras of Ecuador and Peru is described and illustrated. The new species is geographically widely separated from its likely closest relative, *D. yutajensis*, which is endemic to the Guiana Shield region of southern Venezuela and adjacent Brazil, and notably differs in leaf morphology. Vegetative (i.e., epidermal micropapillae, trichomes) and reproductive (i.e., cymule glands, flowers, pollen) micromorphological features were examined with SEM. Rare tristaminate flowers were documented among the typical bistaminate ones. Seeds and diagnostic features among the four species of *Dendrothrix* are compared.

## Introduction

One of the most challenging groups of Euphorbiaceae with regard to identification and classification is the hippomanoid clade of subfamily Euphorbioideae. The clade presently includes 32–40 genera and 300 species grouped in three tribes (Hippomaneae, Hureae, and Pachystromateae; Esser 2001, 2012) or a single large one (Hippomaneae s.l., Webster 2014). Their taxonomy still needs considerable revision, especially at the generic level, in light of molecular phylogenetic results that reveal multiple non-monophyletic groups (Wurdack et al. 2005; Wurdack, unpublished). *Dendrothrix* Esser is one of the many small genera of hippomanoids (19 of the 40 genera contain three or fewer species) that have been relatively recently recognized, and one whose systematics had been confused with other taxa in the clade including *Sapium* Jacq. and *Senefelderopsis* Steyerm. (Esser 1993, 1995). The shared similarities with *Sapium* include their bistaminate flowers with a partly fused calyx, and with *Senefelderopsis* their shared inflorescence structure and Guiana Shield distribution.

**Figure 1.**
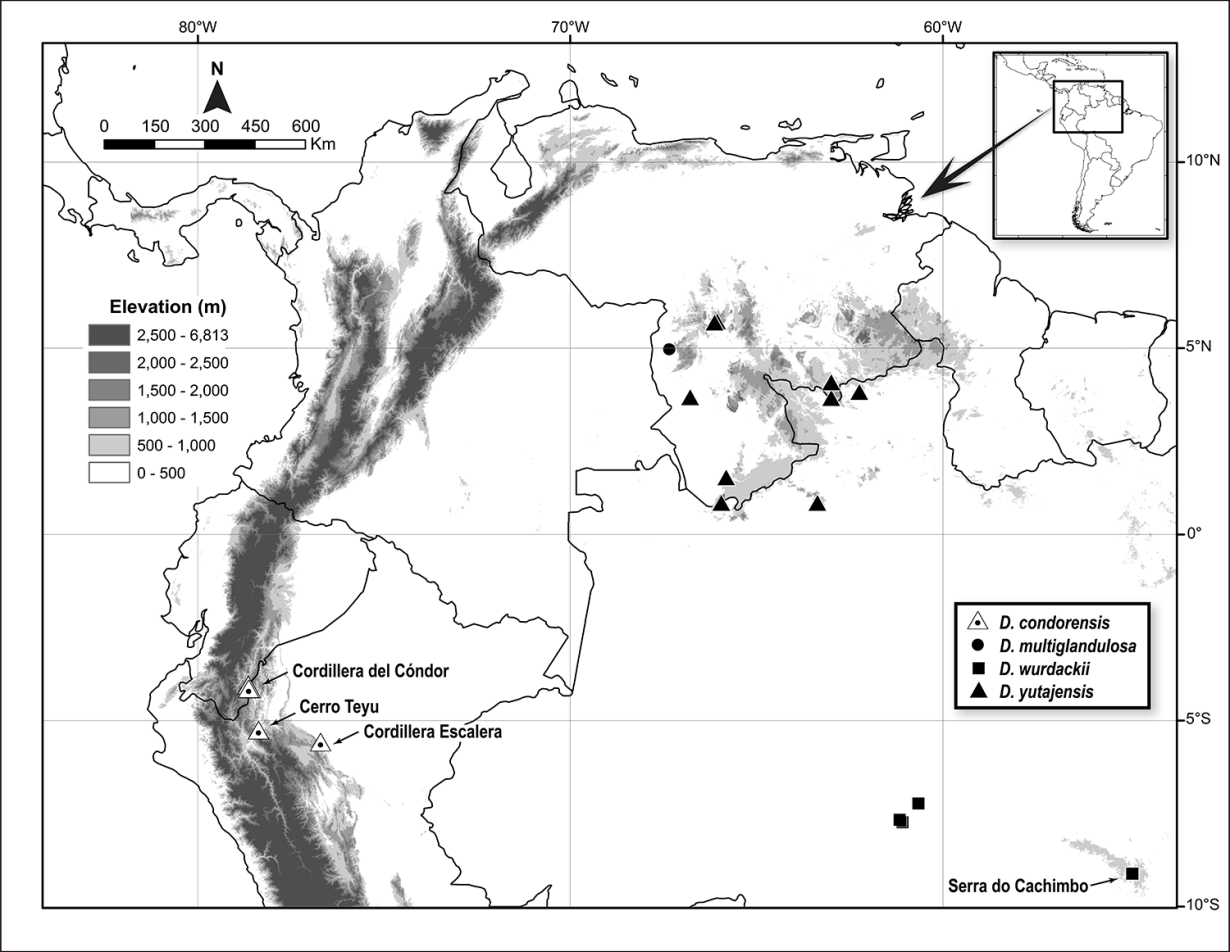
Distribution map of *Dendrothrix* in northern South America.

*Dendrothrix* contains four species of trees and shrubs with white latex, tiny apetalous flowers, and an unusual distribution in northern South America that includes tepuis in the Guiana Shield region and two disjunct outliers (Fig. 1). The genus is distinguished within the hippomanoids by a combination of relatively rare character states including compound thyrse inflorescences, dendritic trichomes, large disc- or cup- shaped bract glands, staminate flowers with two connate stamens, and small, dry fruits. Since Esser’s original studies (1993, 1995) little new information has been published about *Dendrothrix* except for wood anatomy (Mennega 2005). The relationships of *Dendrothrix* were thought to lie with *Senefelderopsis*, and although the former is a distinctive group, Webster (2014) suggested that it might be reduced to a section of *Senefelderopsis*. Recent work on its molecular phylogenetic placement (Wurdack, unpublished) has shown that they are not sister groups and should not be merged. Moreover, a close relationship with *Mabea* Aubl., which had been suggested based on the shared multicellular dendritic trichomes from which *Dendrothrix* gets its generic name (Esser 1993), was also not supported.

*Dendrothrix yutajensis* (Jabl.) Esser was reported for the flora of Ecuador (Neill and Ulloa Ulloa 2011), which represented a considerable range extension. After further study of *Dendrothrix* collections from sub-Andean cordilleras of southern Ecuador and adjacent Peru, I have concluded that they are morphologically distinctive enough to deserve recognition as the new species described herein. These mountains along the wet northeastern flank of the Andes, also referred to as “Andean tepuis,” are rich in endemic taxa, especially in the Cordillera del Cóndor (Neill 2005). Floristic inventory work begun there in the 1990s has yielded numerous new taxa in diverse families. For the Cordillera del Cóndor in particular, its location, climate, and unusual geology of mid-Cretaceous Hollin Formation sandstone in tepui-like mesas support biogeographic affinities with the Guiana Shield biota to the east on pre-Cambrian sandstones, e.g., *Phainantha* Gleason, Melastomataceae (Ulloa Ulloa and Neill 2006); *Stenopadus* S. F. Blake, Asteraceae (Pruski 1998). The new *Dendrothrix* adds the first member of Euphorbiaceae to the growing list of floristic disjunctions between the Andean tepuis and their counterparts in the Guiana Shield. The Euphorbiaceae diversity for the Cordillera del Cóndor region otherwise mostly appears typical of that seen in other montane Andean floras and is not especially rich, with less than 20 taxa. However, among those taxa are the unusual endemic *Croton condorensis* Riina & Cerón (Riina et al. 2014), and the more widely distributed new genus *Incadendron* (Wurdack and Farfan-Rios 2017).

## Taxonomic treatment

### Dendrothrix condorensis

Taxon classificationPlantaeMalpighialesEuphorbiaceae

K.Wurdack sp. nov.

urn:lsid:ipni.org:names:77165931-1

Figure 2

#### Diagnosis.

Differs from *Dendrothrix yutajensis* in larger, thinner leaves with more secondary veins, percurrent tertiary venation, acuminate leaf apex, and seeds lacking a caruncle.

#### Type.

ECUADOR. Zamora-Chinchipe: Nangaritza. Cordillera del Cóndor region, upper Río Nangaritza valley, along road 14.5 km south of Guayzimi, above west bank of Río Nangaritza, sandstone slope with relatively low forest, canopy 20 m, forest being cleared for mining of silica sand for glass manufacture, 04°02'27"S, 78°38'44"W, 930 m, 25 Nov 2005 (fl, fr), *D. Neill & W. Quizhpe 14939* (holotype QCNE; isotypes: AAU, GB, HUT, K, LOJA, MO-6106711, NY, US).

#### Description.

*Shrub* or small tree to 8 m tall, monoecious; stems of leafy branchlets 2–3 mm dia., bark smooth, pith soft. *Exudate* present, white latex. *Indumentum* of multicellular trichomes to 0.1 mm long, ramified, dark reddish-brown. *Leaves* alternate, petiolate, stipulate, simple. *Stipules* free, paired, scale-like, 0.5 × 0.3 mm (width at base), triangular, persistent, eglandular. *Petioles* 40–65 × 1–2.5 mm (dia. mid-length), terete, adaxially slightly canaliculate towards distal end, petiolar glands absent. *Leaf blades*: laminar size class mesophyll, blade 14.5–19.8 × 6.5–8 cm, length:width ratio 2.00–2.98 (mean = 2.63, n = 11), symmetrical, shape elliptic, apex angle acute, apex shape short acuminate to attenuate, base angle acute, base shape cuneate to obtuse; margin entire, slightly revolute; blades sparsely pubescent when mature (young leaf not seen), primary and secondary veins more densely so; lamina thin and brittle when dry, adaxial surface smooth except for prominulous venation, abaxial surface pale, dull due to minute papillae; basilaminar glands present, abaxial, one on each side of primary the vein at the attachment of petiole and hidden under minutely auricled extension of lamina, narrowly elliptic, 1.4–1.8 × 0.4 mm, shallowly sunken into laminar surface and without raised edge, gland surface smooth; embedded laminar gland-like structures (perhaps necrotic, see Discussion), 0–6 per leaf, abaxial, scattered but usually adjacent to secondary or tertiary veins in distal portion of lamina, circular to widely elliptic, 0.5 × 0.3–0.4 mm. *Venation* pinnate, brochidodromous, 18–22 secondaries per side, spacing mostly regular, secondary vein angle gradually increasing along the series from ca 0° for proximal secondaries to 30° for distally diverging veins, course usually straight until curving for distal <1/4 of length, insertion shortly decurrent; intersecondaries parallel to secondaries, infrequent (1–5 per side); intercostal tertiaries alternate percurrent (occasionally opposite); primary to tertiary veins slightly prominulous on both surfaces. *Inflorescence* terminal, erect, compound thyrse, 21–31 cm long, with 2–3 orders of branching, axes moderately pubescent; leaves at base of inflorescence often of reduced size; lateral branches usually bisexual (rarely staminate) with pistillate flowers proximal and staminate distal, branch nodes subtended by usually eglandular bract (rarely at more distal nodes with pair of glands similar to those on cymules); bract acuminate, 2–2.5 × 0.5–0.7 mm (width at base). *Staminate cymules* spirally arranged, 20–22 per lateral inflorescence branch, subtended by bract and pair (1 on each side of bract base) of elongate disc- or cup-shaped glands; bract to 1 (deep) × 2 mm (wide), elliptic, margin sparsely ciliate; glands of proximal cymules 1.2–2 × 0.8–1.5 mm, becoming smaller at distal cymules (unclear if the size decrease is due to serial reduction or decreasing maturity), fleshy, 0.1 mm thick when dried, surface smooth and without pores; bracteoles absent. *Staminate flower* buds 10–16 per cymule, tightly clustered, erect when emergent from subtending bract, globose, to 0.7 mm diameter just before opening; anthetic flowers articulate at base, shortly pedicellate on persistent pedicel to 0.5 mm long; sepals 2 (rarely irregularly 3), connate at base to 0.3 mm, distal lobes 0.4–0.6 × ca 0.5 mm, margin sparsely ciliate; stamens 2(–3), 1–1.5 mm long, barely protruding beyond calyx at anthesis; filaments connate, 0.8–1 × 0.1 mm; anthers 0.4–0.5 mm long, bithecate, apicifixed to subapicifixed with very short connective and pendulous thecae, longitudinally dehiscent via slit 1/2–2/3 length of thecae; pistillode absent; flowers yellow in life. *Pistillate flowers* solitary at 1–2 proximal nodes of lateral thyrse branches; subtending bract 2–2.5 × ca 1.5 mm (width at base), acuminate; bract glands present (distal flower) or reduced to absent (proximal flower), similar to those subtending staminate cymules; short pedicellate, pedicel 0.5–0.8 × 0.5–0.6 mm; flower 3.5–4 mm long; sepals 3, 2–2.5 × 1 mm, free to minutely connate at base (to 0.2 mm), cymbiform, narrowly acute tip, sparsely pubescent, margin sparsely ciliate; ovary 3-locular, ovoid, 1 × 1 mm, top tapering, densely pubescent, distinguished from styles by change in pubescence density; styles connate into trigonous column 1.5–2 × 0.5–0.7 mm, sparsely pubescent; stigmas 3, undivided, slightly flattened, 0.7–1.1 (long) × 0.3 (thick) × 0.4 mm (wide at base), recurved to coiled at anthesis, surface coarsely papillose; placentation apical pendulous with a single ovule per locule; staminodes absent. *Infructescence* consisting of primary axis with lateral fruiting nodes, distal staminate portions of lateral branches deciduous; fruit pedicels 1–2(3) × 0.5–0.7 mm; bracts persistent. *Fruit* subglobose, 5 × 7 mm, sparsely pubescent, apex trilobed due to sunken stylar region, ventral (septal) sutures sulcate, dorsal (loculicidal) sutures with slight ridge; mericarps equal, 2-valved, splitting septicidally then loculicidally to release seeds; sepals, styles, and stigmas persistent. *Pericarp* dry, woody, mericarp wall 0.3 mm thick (equatorial at dorsal suture); exocarp extremely thin (ca 0.05 mm) but locally thickened to 0.2 mm along ventral suture, adherent to mesocarp on dehiscence; mesocarp woody, in equatorial section varying from 0.3 mm thick at dorsal suture to 0.7 mm toward ventral suture; septa woody, nearly continuous except for distal semicircular gap where traversed by funicle, 0.7–1 (wide) × 0.3–0.4 mm (deep); mericarp valves (cocci) remaining attached together after dehiscence via basal triangle 1 × 1–1.3 mm (width at base), slightly twisted when dehisced; septa of mericarps with one bifurcate vascular strand; funicle short, stout, 0.3 × 0.3 mm; columella (carpophore) persistent, 5 × 0.45–0.5 mm (width at narrowest point in middle), dilating to 1–1.5 mm at both tip and base, trigonous, narrowly alate. *Seeds* 3 per fruit, dry, ellipsoid, 4 (long) × 2.7 (wide; lateral-lateral) × 2 mm (deep; raphe-antiraphe); apex with short beak, flattened or depressed around hilar zone, ventral face with shallow groove along which raphe runs as ca. 0.1 mm wide prominulous line; testa dry, smooth, uniformly dark brown, thin (ca 0.05 mm thick); caruncle absent; embryo not seen.

#### Etymology.

The specific epithet refers to the Cordillera del Cóndor, where the type was collected. The mountain range name in turn comes from “condor” based on “kuntur” (Quechua) and refers to the Andean condor (*Vultur gryphus* L.), an important part of the ecology and culture of the Andes.

#### Distribution and ecology.

The new species mostly occurs at 800–1000 m in dense, low, wet forest and sclerophyllous scrub over nutrient poor, acidic, sandstone-derived soils. Such habitats resemble those in the Guiana Highlands occupied by *D. yutajensis*. The three well-separated localities (Cordillera del Cóndor, Cordillera Escalera, Cerro Teyu; Fig. 1) are discoveries due to recent exploration in remote sub-Andean cordilleras, and continued floristic work is likely to extend its range to similar habitats further south in Peru. Floristic affinities between the Cordillera del Cóndor and Cordillera Escalera are notable (Neill et al. 2014). Flowers and fruits were collected during September–November and flowers in March (also fruits in July fide *Croat 91402*, not seen). *Dendrothrix yutajensis* with more collections to finely document phenology, is reproductive from November to May and appears to flower and fruit continuously during this period.

#### Conservation status.

Following the criteria and categories of IUCN (2012), *D. condorensis* is given a preliminary status of Vulnerable (VU) under geographic range criteria B2 area of occupancy <2000 km^2^ (B2a, known to exist at no more than 10 locations; B2b, continuing decline projected). Threats to this taxon in the Cordillera del Cóndor include mining for the underlying silica sand. The Cordillera Escalera is protected as a conservation area.

#### Additional collections.

**ECUADOR. Zamora-Chinchipe**: Nangaritza, Cordillera del Cóndor region, west side of upper Río Nangaritza, along road about 13 km south of Guayzimi, silica mine “La Daniela”, dense wet forest on sloping Hollín sandstone plateau, being mined for silica sand for glass manufacture, 04°08'35"S, 78°35'45"W, 970 m, 15 Sep 2007 (fl.), *D. Neill, C. Davidson, S. Christoph & W. Quizhpe 15747* (AAU, LOJA, MO, NY, QCNE). [Same locality], 15 Sep 2007 (fr.), *D. Neill, C. Davidson, S. Christoph & W. Quizhpe 15750* (AAU, GB, LOJA, MO, NY, QCNE, US). Along road from near Paquisha south to Las Orchídeas, and end of river at Río Nangaritza, via Guayzimi, beginning at 15.9 km E of Zumbi and Río Zamora, then 37.3 km S of junction, 12.3 km N of Las Orchídeas, 04°08'25"S, 78°38'31"W (-4.1402700, -78.6419400), 886 m, 17 July 2004 (fr), *T. Croat, L. Hannon, G. Walhert & T. Katan 91402* (MO; not seen but tentatively included here based on TROPICOS record). **PERU. Amazonas**: Bagua District, upper slopes and summit of Cerro Teyu, summit with sclerophyll scrub, 05°15'56"S, 78°22'07"W, 1030 m, 22 Mar 2001 (fl.), *H. van der Werff, R. Vasquez & B. Gray 16331* (MO, US). **Loreto**: Alto Cahuapanas, Campsite #3 (“Alto Cahuapanas”) on Rapid Biological and Social Inventory #26, -5.66438889, -76.839, 1000–1350 m, 28 Sep 2013, *M. Ríos Paredes 3480* (F). [Same locality], 29 Sep 2013, *M. Ríos Paredes 3517* (F).

#### Discussion.

The four species of *Dendrothrix* are morphologically similar, and major differences are presented in Table 1. *Dendrothrix condorensis* and *D. yutajensis* are likely closely related as they have nearly identical floral features. However, they are easily distinguished by foliar characters (i.e., size, shape, thickness, and details of leaf architecture), seed caruncle differences, and biogeography. Although leaf architecture has not been rigorously compared through clearings and anatomy, several orders of the varyingly prominulous venation are evident in unprepared specimens that allow gross comparisons such as were shown by Esser (1993) to be informative for the group. In *D. condorensis*, secondaries are 18–22 pairs, mostly straight with few course deflections, and distally curving upward for <1/4 of course; intersecondaries are rare, and tertiaries are percurrent (Fig. 3G). In *D. yutajensis*, secondaries that are fewer (usually 7–9 pairs), not straight due to a slight zigzag (fractiflexuous) appearance where tertiaries diverge, and distally curving upward for half of their course; intersecondaries and frequent, and tertiaries are reticulate (Fig. 3J). Even on unusually robust specimens of *D. yutajensis* (e.g., *Maguire & Maguire 35103*, US), the largest leaves (16.5–18 × 6.5–7.8 cm) have only up to 11 pairs of secondaries. The leaves of *D. condorensis* more closely resemble those of *D. multiglandulosa* Esser with which it shares the generally larger blades and percurrent venation. *Dendrothrix multiglandulosa* is only known from two collections from the Cuao-Sipapo massif in Venezuela and potential variation is poorly understood. *Dendrothrix yutajensis* is the most frequently collected and wide-ranging species of *Dendrothrix*, known from many well-separated tepuis, but intraspecific variation appears low. *Dendrothrix wurdackii* Esser has previously unrecognized intraspecific variation with an odd collection (i.e., BRAZIL, Pará: km 872 Cuiabá-Santarém [Highway], Serra do Cachimbo, forest beside small stream, 6 Nov 1977, *G. Prance 24947*, MO, NY, US), having much larger (to 18 × 8.5 cm), long acuminate, glabrous leaves with poorly developed abaxial micropapillae as compared with the four other collections of the species that closely resemble the type. This collection, which Grady Webster annotated as a new species of *Senefeldera* Mart., clearly has closest affinities with *D. wurdackii* based on similarities in leaf shape and venation, basilaminar glands, pistillate sepal shape, and biogeography. While the morphological differentiation and slight disjunction (Fig. 1) may be significant, it is not to the degree used here to justify the recognition of *D. condorensis* and I have refrained from further taxonomic adjustments until *D. wurdackii* is better understood.

Leaf micromorphological features are similar among the taxa of *Dendrothrix*. The distinctive, loosely attached, branched trichomes (Fig. 3E, K) are structurally very similar but can differ in pigmentation (rusty brown versus whitish; see Table 1). Epidermal microrelief such as micropapillae or striations that give an often light-colored, matte sheen to abaxial leaf surfaces has evolved in many genera of Euphorbiaceae, including *Dendrothrix*, and other hippomanoids (e.g., *Gymnanthes hypoleuca* Benth., *Sebastiania larensis* Croizat & Tamayo, *Senefelderopsis croizatii* Steyerm.). The microrelief likely has functional significance related to optimized stomatal conductance and reduced leaf wetting (Neinhuis and Barthlott 1997). The morphology of the leaf micropapillae in *Dendrothrix* is unusual and may be synapomorphic. They have positional size variation in being short to absent on major veins and grading to longer in intercostal regions (Fig. 3K). In *D. condorensis*, the micropapillae are finger-like, 25–30 μm tall, ornamented with ridges, and form a protective canopy that obscures the stomata (Fig. 3H–I). Epicuticular waxes are not well developed, in keeping with the non-glaucous appearance. The stomata are only abaxial, with the adaxial surface being relatively featureless (Fig. 3I). Glands are often present on leaves and/or inflorescences of Euphorbiaceae, and *Dendrothrix* is no exception where they occur in pairs at the base of the leaf and subtending the bracts. The nature of scattered laminar glands in *Dendrothrix* is partly unclear. In the case of *D. wurdackii* they resemble typical Euphorbiaceae laminar glands with a well defined raised border and an abaxial distribution adjacent to secondary veins towards the leaf margin. The glands on *Prance 24947* contain drops of clear exudate that are still sticky after 40 years. I have not found clearly homologous structures on the other taxa of *Dendrothrix*. Circular to widely elliptic, small (<0.5 mm diameter), gland-like structures with smooth surfaces and without raised edges are sparsely present in *D. condorensis* and more abundantly so in *D. multiglandulosa*, where in the latter they were considered of systematic significance (Esser 1993). Although not examined anatomically, these appear to be wound response to microbial damage rather than typical glands as evidenced by their absence in the few young leaves available, erratic distribution, the presence of apparently intermediate epidermal blistering stages that are clearly necrotic, and margins that are not clean under SEM (not shown) but rather contain fragments that suggest micropapillae residue from surficial degradation.

Among reproductive characters that are variable among the species are details of the pistillate flowers including sepal and stigma morphology (see Table 1). Staminate bract glands vary in number with *Dendrothrix multiglandulosa* usually having 2–3 pairs (variable from 1–4, and paired or unequal in number between the bract sides), while the other species have one pair (*D. yutajensis* rarely has a second gland on one side, e.g., *Maguire & Maguire 35103*, NY). Inflorescence branching is potentially variable, with 2–3 orders for *D. condorensis* and *D. multiglandulosa*, and the other species have only two orders. It is unclear if this represents noteworthy interspecific variation or possible collector bias for smaller inflorescences that better fit herbarium sheets. *Dendrothrix* is characterized by staminate flowers with two connate anthers (Fig. 4D–E). Rare flowers with three anthers are present on collections of *D. condorensis* (i.e., *Neill & Quizhpe 14939*, MO) and *D. yutajensis* (i.e., *Maguire & Maguire 35103*, NY). Such flowers have more or less symmetrical androecia, connate filament columns, partial third sepals, and developed pollen, which indicates they may be functional despite being teratological in gross structure (Fig. 4F). The triandrous flowers may be of significance in reflecting the likely plesiomorphic condition for the tribe and the ease of merosity change even in groups that appear fixed. The bract glands are smooth and without pores (Fig. 4B–C). The staminate sepals bear stomata and are externally minutely papillose (Fig. 4A). The pollen of *Dendrothrix* (Fig. 4G; Esser 1994) is tricolporate with a perforate exine, which closely resembles that of other Hippomaneae (see Park and Lee 2013). Seed variation occurs among the taxa of *Dendrothrix* (Fig. 3A–D) with a wider, more globose seed in *D. multiglandulosa*, and a well-developed caruncle in *D. wurdackii*. In *D. yutajensis* the caruncle is small, and there is no caruncle in *D. condorensis* or *D. multiglandulosa*. The 13 available seeds of *D. condorensis* from dehisced fruits (some slightly immature; note lighter brown coat in Fig. 3B relative to other samples) do not show evidence of caruncle growth (Fig. 3F), while all seeds that I examined of *D. yutajensis* have a small caruncle.

*Dendrothrix* has a noteworthy Guiana Shield disjunct distribution (Fig. 1), which now combines two patterns with its over 1000 km distant outliers including elements in both the Andean (i.e., *D. condorensis*) and Amazon (i.e., *D. wurdackii*) phytogeographic regions. Such a tripartite distribution does not appear to be shared with other Guiana Shield near-endemic plant genera, although many have disjunct species in just one of those regions (Berry and Riina 2005). *Raveniopsis* Gleason (Rutaceae) also has a distribution of Guiana Shield endemics and two Brazilian Amazon lowland outliers, and interestingly, one of the few localities of *D. wurdackii* (*Calderón et al. 2682*, NY) is also the type locality for one of those outliers, *Raveniopsis campinicola* Kallunki (*Calderón et al. 2722*, US).

**Figure 2.**
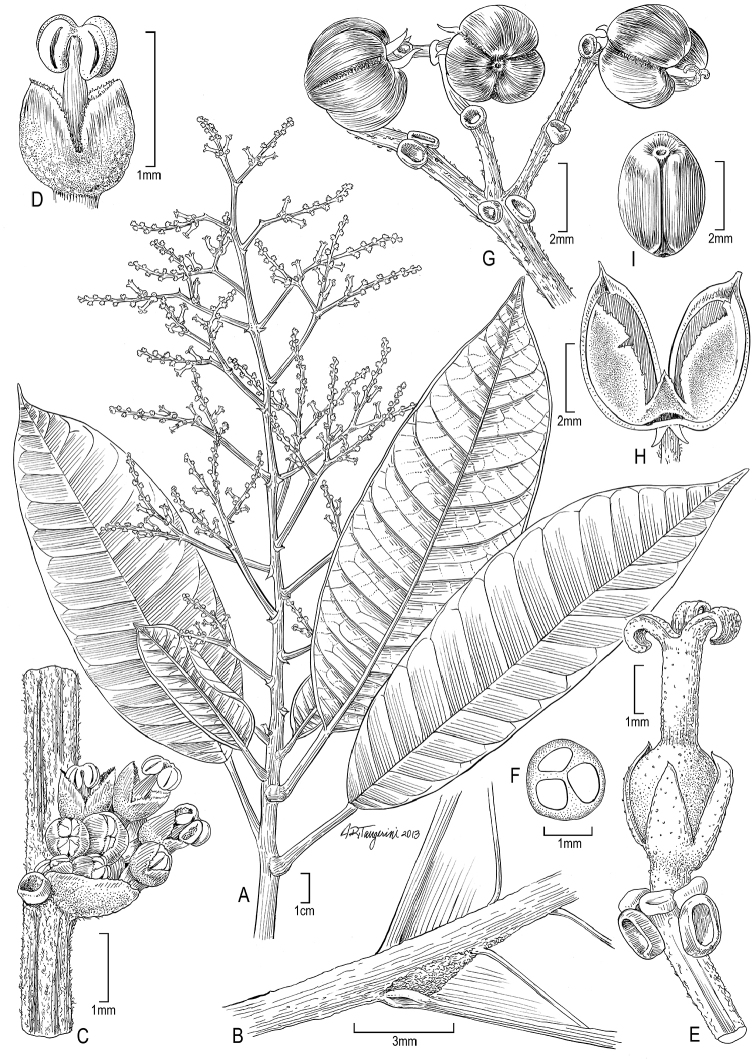
Illustration of *Dendrothrix condorensis*. **A** Habit **B** Leaf base (abaxial) showing basilaminar glands **C** Staminate cymule **D** Staminate flower **E** Pistillate flower **F** Cross section of ovary **G** Portion of infrutescence **H** Mericarp valve **I** Seed. (Source: **A** *Neill et al. 15747*, MO and using life photos of this collection; **B, E–F** *van der Werff et al. 16331*, MO; **C–D, G** *Neill & Quizhpe 14939*, MO; **H–I** *Neill et al. 15750*, MO).

**Table 1.** Diagnostic features to distinguish among the species of *Dendrothrix*.

Character	*D. condorensis*	*D. yutajensis*	*D. multiglandulosa*	*D. wurdackii*
Leaf features	Blade elliptic, 14.5–19.8 × 6.5–8 cm, apex acuminate to attenuate, base cuneate to obtuse; secondary veins 18–22 pairs, straight; tertiary veins mostly alternate (occasionally opposite) percurrent; texture thin and brittle	Blade elliptic, 7-12.5(18) × 3.5–6.5(7.8), apex acute to subobtuse, base cuneate to obtuse; secondary veins 7–9 (11) pairs, flexuous; tertiary veins reticulate; texture coriaceous	Blade elliptic to obovate, 13.5–16.5 × 5.5–9 cm, apex acute to obtuse, base cuneate to obtuse; secondary veins 9–14 pairs, straight; tertiary veins mostly alternate (rarely opposite) percurrent; texture coriaceous	Blade ovate, 8–10 × 6–7 cm, apex acute to acuminate, base subcordate to rounded; secondary veins 5–9 pairs, including prominent basal pair of major secondaries such that the leaf appears triplinerved, straight; tertiary veins reticulate; texture coriaceous
Basilaminar glands	Abaxial, hidden under leaf extension	Abaxial, hidden under leaf extension	Abaxial, hidden under leaf extension	At leaf margin and sometimes nearly adaxial (never hidden by laminar extension)
Pubescence	Reddish brown	Reddish brown	Pale, whitish	Reddish brown
Staminate cymule bract glands	1 per side	1(2) per side	(1)2–3(4) per side	1 per side
Pistillate sepals	Free to minutely connate at base (to 0.2 mm), lobes narrowly acute; margin entire, sparsely ciliate	Free to minutely connate at base (to 0.1 mm), lobes narrowly acute; margin entire, pubescent/ciliate	Distinctly connate at base (to 0.8 mm), lobes rounded to broadly acute; margin entire, ciliate	Distinctly connate at base (to 0.5 mm), lobes rounded to broadly acute; margin erose or irregularly minutely toothed, very sparsely ciliate
Pistil	Style 1.5–2 × 0.5–0.7 mm; stigma branches long (to 1.1 mm), thin, recurved/coiled	Style 1.5(–2) × 0.5–0.7; stigma branches long (to ca 1 mm), thin, recurved/coiled	Style 1–1.3 × 0.5–0.7 mm; stigma branches short (to 0.5 mm), spreading but not recurved	Style 1–1.5 × 0.5–0.6 mm; stigma branches long (to ca 1 mm), thin, recurved/coiled
Caruncle	Absent (*Neill 15750*, MO; *Neill & Quizhpe 14939*, MO, although more immature)	Present, small (*Maguire 30694*, NY; *Nee 31120*, NY, US; *Amaral 1523*, MO)	Absent (*Maguire & Politi 27683*, NY)	Present, large (*Calderón et al. 2682*, US; Esser 1993)
Distribution	Ecuador (Zamora-Chinchipe), Peru (Amazonas, Loreto)	Brazil (Amazonas), Venezuela (Bolívar, Amazonas)	Venezuela (Amazonas)	Brazil (Amazonas, Pará)

**Figure 3.**
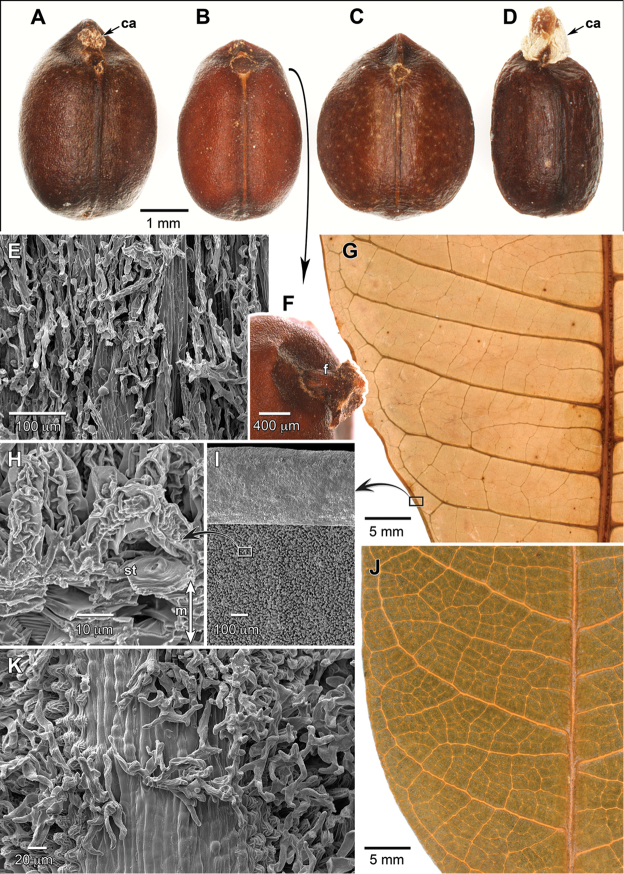
Seeds and surfaces of *Dendrothrix*. **A–D** Ventral views of seeds (ca = caruncle) **A** *D. yutajensis* **B** *D. condorensis* **C** *D. multiglandulosa* **D** *D. wurdackii* **E** *D. condorensis*, inflorescence axis showing dendritic trichomes **F** *D. condorensis*, top of seed showing short funicle (f) and caruncle absence **G** Abaxial view of *D. condorensis* leaf **H** *D. condorensis* transverse fractured leaf showing abaxial papillae and stomata (st), above intercellular space in the spongy parenchyma (m = mesophyll) **I** *D. condorensis* abaxial leaf micropapillae contrasting with smooth revolute adaxial leaf margin at image top **J** Adaxial view of *D. yutajensis* leaf **K** *D. wurdackii*, abaxial leaf surface along secondary vein showing gradient of micropapillae development at vein edges, and overlying dendritic trichomes. (**A–D, F–G, J** imaged with a Olympus DSX100 **E, H–I, K** imaged with a Zeiss EVO MA15 SEM at 10–12 kV after sputter coating with 25 nm of Au/Pd; SEM samples untreated and directly mounted from dried herbarium specimens. Source: **A** *Nee 31120*, NY; **B, F** *Neill et al. 15750*, MO; **C** *Maguire & Politi 27683*, NY; **D** *Calderón et al. 2682*, US; **E, G–I** *Neill & Quizhpe 14939*, MO; **J** *Amaral 1523*, MO; **K** *Cid Ferreira 5797*, NY).

**Figure 4.**
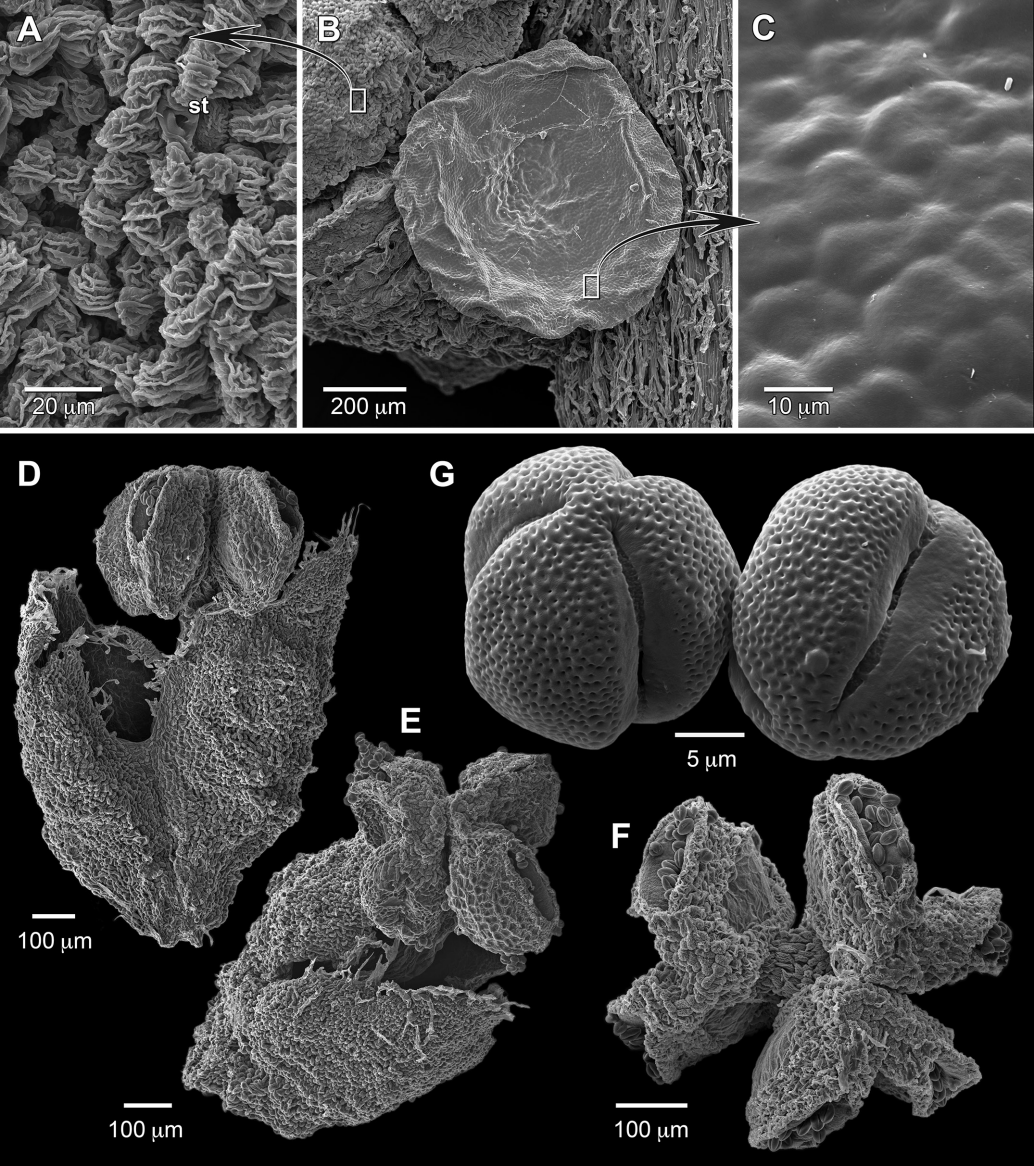
Floral morphology of *Dendrothrix condorensis*. **A** Staminate sepal outer surface showing papillae and stomata (st) **B** Staminate cymule base showing subtending discoid gland **C** Surface of staminate cymule gland **D** Staminate flower **E** Staminate flower **F** Triandrous flower, top view of slightly asymmetrically fused androecium **G** Pollen. (Source: SEM of *Neill & Quizhpe 14939*, MO).

## Supplementary Material

XML Treatment for Dendrothrix condorensis
